# Causal Interactive Links Between Presence and Fear in Virtual Reality Height Exposure

**DOI:** 10.3389/fpsyg.2019.00141

**Published:** 2019-01-30

**Authors:** Daniel Gromer, Max Reinke, Isabel Christner, Paul Pauli

**Affiliations:** ^1^Department of Psychology, Biological Psychology, Clinical Psychology and Psychotherapy, University of Würzburg, Würzburg, Germany; ^2^Center of Mental Health, Medical Faculty, University of Würzburg, Würzburg, Germany

**Keywords:** presence, fear, virtual reality, visual realism, acrophobia

## Abstract

Virtual reality plays an increasingly important role in research and therapy of pathological fear. However, the mechanisms how virtual environments elicit and modify fear responses are not yet fully understood. Presence, a psychological construct referring to the ‘sense of being there’ in a virtual environment, is widely assumed to crucially influence the strength of the elicited fear responses, however, causality is still under debate. The present study is the first that experimentally manipulated both variables to unravel the causal link between presence and fear responses. Height-fearful participants (*N* = 49) were immersed into a virtual height situation and a neutral control situation (fear manipulation) with either high versus low sensory realism (presence manipulation). Ratings of presence and verbal and physiological (skin conductance, heart rate) fear responses were recorded. Results revealed an effect of the fear manipulation on presence, i.e., higher presence ratings in the height situation compared to the neutral control situation, but no effect of the presence manipulation on fear responses. However, the presence ratings during the first exposure to the high quality neutral environment were predictive of later fear responses in the height situation. Our findings support the hypothesis that experiencing emotional responses in a virtual environment leads to a stronger feeling of being there, i.e., increase presence. In contrast, the effects of presence on fear seem to be more complex: on the one hand, increased presence due to the quality of the virtual environment did not influence fear; on the other hand, presence variability that likely stemmed from differences in user characteristics did predict later fear responses. These findings underscore the importance of user characteristics in the emergence of presence.

## Introduction

Psychological treatments using virtual reality (VR) have shown promising results for different psychopathologies (Riva et al., 2016; Freeman et al., 2017), including specific phobia (Shiban et al., 2017; Meyerbröker et al., 2018), social phobia (Bouchard et al., 2017), PTSD (Rothbaum et al., 2014; Beidel et al., 2017), eating disorders (Manzoni et al., 2016; Ferrer-García et al., 2017), and schizophrenia (du Sert et al., 2018; Pot-Kolder et al., 2018), among others. To date, the most evidence for the efficacy of VR treatments has been shown in phobic disorders (Parsons and Rizzo, 2008; Powers and Emmelkamp, 2008; Opris et al., 2012; Turner and Casey, 2014; Morina et al., 2015), where pioneering studies established VR as a treatment medium as early as the late 1990s (e.g., for claustrophobia treatment, Botella et al., 1998). In virtual reality exposure therapy (VRET) for specific phobias, VR is used to simulate threatening environments and stimuli (e.g., virtual heights), allowing to expose patients to their fear (Riva et al., 2016). The ability of virtual environments (VE) to elicit symptoms of pathological fear has been shown in numerous studies (Diemer et al., 2014), yet the factors influencing how much fear is elicited (Diemer et al., 2015) and how phobic stimuli should be presented for optimal outcome of VRET (Freeman et al., 2017) are still not fully understood.

The VR-related psychological construct of *presence*, the user’s sense of ‘being there’ in the VE, is widely assumed to be crucial for the fear responses in VR (Slater et al., 1994; Witmer and Singer, 1998; Cummings and Bailenson, 2016). Regarding the relationship between presence and therapy efficacy, Freeman et al. (2017, p. 2394) for example state that “VR has extraordinary potential to help people overcome mental health problems if high levels of presence are achieved […]”. However, the few studies on the effect of presence on VRET efficacy revealed mixed results (Schuemie et al., 2000; Krijn et al., 2004; Price and Anderson, 2007; Quero et al., 2008; Price et al., 2011). Likewise, a causal relationship between presence in VR and strength of fear responses when exposed to the feared stimulus or situation is assumed. For example, Price et al. (2011, p. 768) state that “[…] presence is the mechanism by which a virtual stimulus can elicit fear […]”. However, the assumed relationship between presence and fear is mainly confirmed by reports of positive correlations between both measures (see Ling et al., 2014, for a meta-analysis). A possible causal relationship has not been demonstrated unequivocally yet and therefore is still subject to debate (Diemer et al., 2015; Peperkorn et al., 2015; Riva et al., 2015).

Few experimental studies tried to demonstrate the assumed causal relationship between presence and fear in VR. Bouchard et al. (2008) compared effects of an anxiety-inducing VE vs. a control VE on ratings of presence and anxiety. They found higher presence ratings in the anxiety-inducing VE and concluded that the increase in anxiety caused the increase in presence. Peperkorn et al. (2015) studied associations between presence and fear by exposing participants multiple times to a virtual spider. They concluded that presence predicted fear (and not the other way around) in early trials, whereas the relationship became bidirectional in later trials. Robillard et al. (2003) exposed phobic and non-phobic participants to phobic stimuli and environments and assessed both presence and anxiety ratings. The authors then conducted stepwise linear regression analyses on both presence and anxiety ratings to find the best predictors for each variable. Since both variables were important predictors of each other, the authors concluded that the results “indicate a synergistic relationship between presence and anxiety” (Robillard et al., 2003, p. 467). Shortcomings of these previous studies are that they were either correlational or they manipulated only one of both variables. To our knowledge, the present study is the first which experimentally manipulated both presence and fear and assessed presence ratings as well as verbal and physiological fear responses.

Fear is typically manipulated by presenting stimuli and environments relevant vs. irrelevant to a given phobia (Bouchard et al., 2008; Alsina-Jurnet and Gutiérrez-Maldonado, 2010) and/or by comparing fear responses of phobic vs. non-phobic participants (Robillard et al., 2003; Alsina-Jurnet and Gutiérrez-Maldonado, 2010). Both approaches are effective, and we followed the former approach by presenting a virtual height and a control environment to height-fearful participants.

Experimental manipulation of presence may be achieved by changing hardware characteristics of the VR system or the quality of the VE. Increased field of view, use of stereoscopy, and increased levels of user-tracking were found to show clear effects on presence (Cummings and Bailenson, 2016). In contrast, manipulations of *sensory realism*, i.e., quality of visual and auditory simulations, had mixed results (Cummings and Bailenson, 2016), although knowing the relevance of these two factors for the experience of presence would be of high interest especially for researchers who develop VEs. According to Christou and Parker (1995), visual realism “can be equated with how closely the artificial world resembles a corresponding possible real world” (Christou and Parker, 1995, p. 53). Elements of visual realism are geometry (e.g., vertex count), lighting (e.g., static vs. dynamic shadows, soft vs. hard shadows) and material properties (e.g., texture resolution, use of normal maps) (Slater et al., 2009; Reinhard et al., 2013). Some studies found increased presence with higher visual realism (Welch et al., 1996; Slater et al., 2009; Kwon et al., 2013), whereas other studies did not find such an effect (Dinh et al., 1999; Zimmons and Panter, 2003; Mania and Robinson, 2004; Lee et al., 2013; Lugrin et al., 2015). Similarly, some studies found a positive effect of auditory simulation (e.g., absence vs. presence of sound, stereo vs. spatial sound) on presence (Hendrix and Barfield, 1996; Dinh et al., 1999; Larsson et al., 2007; Brinkman et al., 2015), while other studies could not find such an effect (Nichols et al., 2000; Keshavarz and Hecht, 2012a,b). Please note that these studies used different manipulations of visual realism and/or auditory content, and also different measures for presence (Kober and Neuper, 2013), and therefore, conclusions about the best option to manipulate presence cannot be drawn. We decided to manipulate presence by changing the sensory realism of VEs because of the high relevance for researchers and because the need to advance unequivocal findings.

The present study exposed height-fearful participants to a fear-eliciting VE versus two neutral control VEs (fear manipulation, within subjects), whereby half of the participants experienced high sensory realism VEs (visual content of high quality and with auditory simulation) versus low sensory realism VEs (visual content of low quality and without auditory simulation) for the other half (presence manipulation, between subjects). Presence as well as verbal and physiological (skin conductance and heart rate) fear responses were registered. Our hypotheses were: (1) higher quality of visual and auditory content of the VE increases presence, and (2) there is a causal relationship between presence and fear responses, i.e., either (2a) increased fear levels (comparing the height vs. the neutral situation) lead to a higher reported sense of presence (fear → presence), or (2b) increased presence (comparing high quality vs. low quality simulations) leads to stronger fear responses (presence → fear).

## Materials and Methods

### Sample

Potential participants were recruited via advertisement and the university subject pool, and were screened for fear of heights using a subset of the Acrophobia Questionnaire (AQ, Cohen, 1977) to predict AQ scores. Volunteers with estimated scores between 20 and 50 (targeting a height-fearful but non-clinical population) were invited to the study and 49 participants (age: *M* = 26.84, *SD* = 10.94; 37 female) were included. The experimental procedure was approved by the Ethics Committee of the Institute of Psychology at the University of Würzburg. All participants gave written informed consent in accordance with the Declaration of Helsinki. Participants received either 8 EUR or course credit for participation.

### Apparatus

The virtual environment was rendered in Unreal Engine 4.12 (Epic Games, Cary, NC, United States) using assets from the Open World Demo Collection and was displayed on a HTC Vive (HTC, New Taipei City, Taiwan) with a resolution of 1080 pixels × 1200 pixels per eye at 90 Hz, and a 100° field of view. The experiment ran on a Windows 10 64-bit machine with an Intel Core i5-6600k, 16 GB RAM and a Nvidia GTX 970. A Sennheiser HD 439 (Sennheiser, Wedemark-Wennebostel, Germany) was used for audio presentation. Physiological signals (electrodermal activity, electrocardiogram) were recorded by a Brainproducts V-AMP 16 and the Vision Recorder 1.2 software (Brain Products, Munich, Germany).

### Experimental Design and Procedure

A 2 × 3 mixed design was used for the study. Experimental manipulations were *presence manipulation* by means of sensory realism (low vs. high, between factor) and *fear manipulation* with different situations (control 1 vs. height vs. control 2, within factor). Participants were randomly assigned to the between subject factor.

**FIGURE 1:**
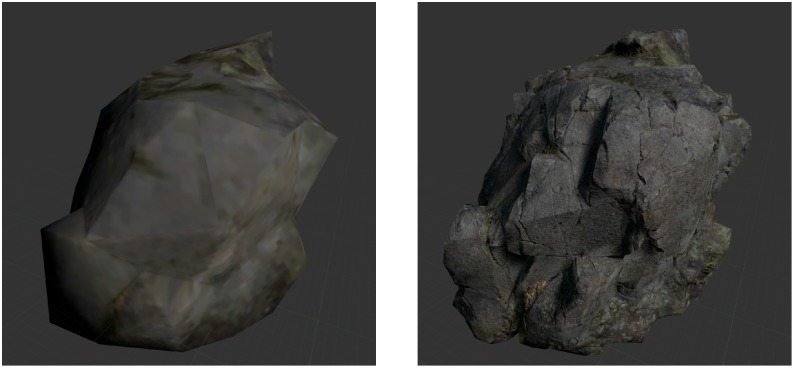
Example for the manipulation of visual realism. In the low and high sensory realism conditions, the rock was rendered with 152 vertices and simplified texture **(left)**, and 2,342 vertices and fine-grained texture **(right)**, respectively.

**FIGURE 2:**
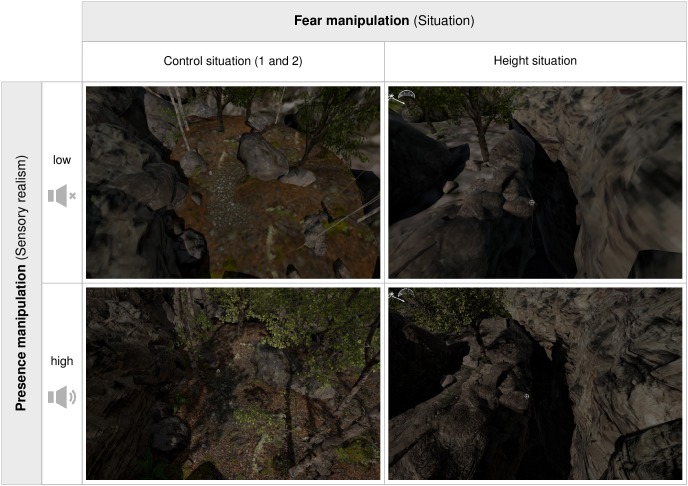
Screenshots of the control situations **(left)** and height situation **(right)** with low **(top)** and high **(bottom)** sensory realism.

**FIGURE 3:**
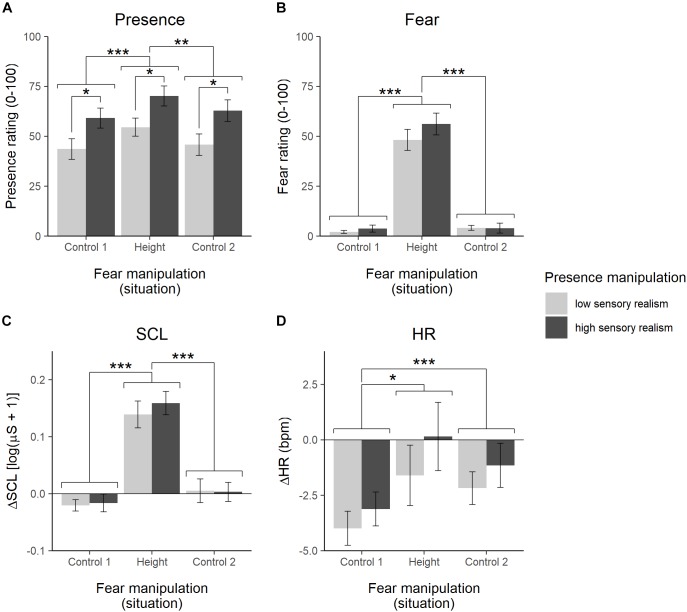
Mean ratings (±standard error) of presence **(A)** and fear **(B)**, and mean changes (±standard error) in skin conductance level (SCL) **(C)** and heart rate (HR) **(D)** differentiated for the three virtual environment conditions (fear manipulation) and the two sensory realism conditions (presence manipulation). ^∗^*p* < 0.05, ^∗∗^*p* < 0.01, ^∗∗∗^*p* < 0.001.

For the fear manipulation, two different environments were created: a control situation which exposed participants to a forest environment surrounded by rocks and trees, and a height situation which exposed participants to the edge of a 30 m deep canyon. These VEs were manipulated regarding sensory realism to induce different levels of presence. This was realized by modifying both the visual realism of the VE as well as the auditory content. The low sensory realism condition was derived from the high sensory realism condition by (1) simplifying polygon meshes by scaling down the vertex count of meshes to 5–10% using the Decimate modifier in Blender, (2) reducing texture quality by applying both a Mosaic filter and Surface Blur filter to the textures in Photoshop (see Figure 1), (3) replacing tree meshes with two-dimensional bitmaps (sprites), and (4) turning sound off (see Figure 2 for demonstration of the different conditions).

After the arrival in the laboratory, participants read and signed the informed consent. Participants were then equipped with electrodes for heart rate and skin conductance measurement. During a baseline measure of 5 min, participants filled in questionnaires (demographics, Acrophobia Questionnaire, and State-Trait Anxiety Inventory) and read an information letter, which described the concept of presence in VR, and how it would be rated during the experiment. Subsequently, participants were placed in the center of the VR tracking area and helped to put on the head-mounted display and headphones. The actual experiment consisted of the fixed sequential exposure to three situations, which were presented in either their high or low sensory realism version: the control situation (control 1), the height situation (height condition), and again the control situation (control 2). Each trial consisted of a fade-in of the virtual scene, a 1-min exploration phase where participants could look around, and a rating phase where participants were asked to give their fear and presence ratings, followed by a fade-out of the virtual scene. After taking off the head-mounted display, participants filled in another set of questionnaires (State-Trait Anxiety Inventory: state anxiety subscale only, Simulator Sickness Questionnaire, and MEC Spatial Presence Questionnaire).

### Measures

#### Questionnaires

*Acrophobia Questionnaire* (AQ; Cohen, 1977) is a self-report questionnaire that assesses trait height anxiety on the subscales anxiety and avoidance. The subscale for anxiety comprises of 20 situational items (α = 0.86), such as “standing next to an open window on the third floor.” Each item is rated on a seven-point Likert Scale ranging from 0 (“not at all anxious”) to 6 (“extremely anxious”), resulting in a sum score of 0–120. The avoidance subscale consists of the same 20 situational items (α = 0.73). Each item is rated on a three-point Likert Scale (“would not avoid doing it,” “would try to avoid doing it,” and “would not do it under any circumstances”), resulting in a sum score of 0–40.

*State-Trait Anxiety Inventory* (STAI; Laux et al., 1981) is a self-report questionnaire that measures state and trait anxiety. The state anxiety subscale consists of 20 items (e.g., “I am calm”) that are rated on a four-point Likert Scale ranging from “not at all” to “very much so” (α = 0.85 at pre- and α = 0.93 at post-measurement, respectively). Participants are asked to rate the statements according to their present feelings. The trait anxiety subscale also consists of 20 items (e.g., “I am content”) which are rated on a four-point Likert Scale ranging from “almost never” to “almost always” (α = 0.92). Participants are asked to rate the statements according to how they feel generally. The range for both scales is from 20 to 80. The STAI was measured as a control variable.

*Simulator Sickness Scale* (SSQ; Kennedy et al., 1993) is a self-report questionnaire that measures simulator sickness, that is symptoms such as nausea, dizziness, headache, or eyestrain, resulting from immersions into VEs. The questionnaire comprises 16 items rated on a four-point Likert Scale ranging from “none” to “severe.” The resulting sum scores are associated with the three factors nausea (e.g., stomach awareness) (α = 0.75), oculomotor problems (e.g., eyestrain) (α = 0.57), and disorientation (e.g., vertigo) (α = 0.78), as well as a total score (α = 0.85). The SSQ was measured as a control variable.

*MEC Spatial Presence Questionnaire* (MEC-SPQ; Vorderer et al., 2004) is a self-report questionnaire that measures different aspects of spatial presence. It builds upon the process model of spatial presence by Wirth et al. (2007) and consists of eight subscales measured by either 4, 6, or 8 items, respectively, rated on a five-point Likert Scale ranging from 1 (“I do not agree at all”) to 5 (“I fully agree”). In the current study, five subscales were used in the 8-item version: *Attention Allocation* (e.g., “I devoted my whole attention to the virtual environment.”) (α = 0.89), *Spatial Situation Model* (e.g., “I had a precise idea of the spatial surroundings presented in the virtual environment.”) (α = 0.84), *Spatial Presence: Self Location* (e.g., “I felt as though I was physically present in the environment of the presentation.”) (α = 0.92), *Spatial Presence: Possible Actions* (e.g., “I had the impression that I could be active in the environment of the presentation.”) (α = 0.89), and *Suspension of Disbelief* (e.g., “I concentrated on whether there were any inconsistencies in the virtual environment”) (α = 0.88). The three remaining subscales *Higher Cognitive Involvement*, *Domain Specific Interest*, and *Visual Spatial Imagery* were not measured because of the length of the full questionnaire and our focus on subscales that measure spatial presence in the narrower sense. The questionnaire therefore comprised 40 items.

#### Online Ratings

Fear ratings were assessed by means of Subjective Units of Discomfort Scales (SUDS) ranging from 0 to 100. Presence ratings were assessed using the question “To which extent did you feel present in the virtual environment, as if you were really there?” (Bouchard et al., 2004) with a range from 0 to 100.

#### Physiological Measures

##### Heart rate (HR)

The electrocardiogram (ECG) was derived using three Ag/AgCl electrodes placed under the right collarbone, on the lower left costal arch (reference electrode), and on the lower left back (ground electrode), recorded at a sample rate of 500 Hz. The ECG was filtered offline with a 50 Hz notch filter and a 2.5 Hz high-pass filter. Detection of R waves and correction of interbeat interval artifacts was done in PeakMan 0.3.0^1^. The sequence of interbeat intervals was processed with the R package phyr6^2^. First, the sequence was segmented (control situation 1, height situation, and control situation 2) and subsequently baseline corrected, using the phase where participants filled in questionnaires as baseline. Second, the mean heart rate change from baseline (ΔHR in bpm) was calculated for each segment.

##### Skin conductance level (SCL)

The electrodermal activity (EDA) was derived using two 13/7 mm Ag/AgCl electrodes filled with 0.5% NaCl gel. The electrodes were placed on the thenar and hypothenar of the right hand and the signal was recorded at a sample rate of 500 Hz. Segmentation was done analogously to the ECG signal. Before applying the baseline correction, the EDA signal was added to 1 and logarithmized to control for skewness. The change in SCL from baseline [ΔSCL in log(μS + 1)] was then calculated by computing the mean of each segment.

### Data Analysis

All statistical analyses were conducted with R 3.5.0 (R Core Team, 2016). The afex package (Singmann et al., 2016) was used for ANOVA with type 3 sum of squares, and the emmeans package (Lenth, 2018) was used for *post hoc* comparisons (using Tukey’s method for alpha adjustment for multiple comparisons). In the ANOVA for presence ratings, one participant had to be excluded due to missing data and in the ANOVA for SCL another participant had to be excluded due to technical problems with the electrodes. The cross-lagged panel model was fitted using the lavaan package (Rosseel, 2012) and displayed with the semPlot package (Epskamp, 2017).

## Results

### Group Characteristics

Participants in the two experimental conditions did not differ in sex, χ^2^ = 0.06, *p* = 0.802, as well as height-fearfulness, state and trait anxiety, and simulator sickness after the experiment (see Table 1).

**Table 1:** Questionnaire data.

	Low sensory realism		High sensory realism			
	*M*	*SD*	*M*	*SD*	*t*	*p*
Age	27.64	11.48	26.00	10.53	0.52	0.605
AQ Anxiety	36.28	14.35	39.09	13.81	−0.68	0.498
AQ Avoidance	7.48	4.82	8.96	3.51	−1.22	0.229
STAI State *t*_1_	35.60	6.53	33.46	5.53	1.24	0.221
STAI State *t*_2_	39.43	10.81	35.52	7.22	1.44	0.157
STAI Trait	37.00	7.36	37.75	10.13	−0.29	0.771
SSQ Total	26.80	30.26	25.56	24.15	−0.16	0.875

*AQ, Acrophobia Questionnaire; STAI, State-Trait Anxiety Inventory (t_1_ = at the beginning and t_2_ = in the end of the experiment); SSQ, Simulator Sickness Questionnaire.*

### Influence of Sensory Realism on Presence

In order to test whether the manipulation of presence by means of sensory realism was successful, a two sample *t*-test on the presence rating in the first control situation was conducted. The test showed a significant difference between sensory realism conditions, *t*(46.93) = 2.31, *p* = 0.026, *d* = 0.66. Participants in the high sensory realism condition (*M* = 60.0, *SD* = 23.9) reported significantly higher presence than participants in the low sensory realism condition (*M* = 43.6, *SD* = 25.9). For the MEC-SPQ scores, a one-way MANOVA with the five subscales as dependent variables and sensory realism as independent variable revealed no main effect of sensory realism, Wilks’ λ = 0.94, *F*(5,41) = 0.56, *p* = 0.726 (see Supplementary Material for descriptive statistics).

### Causal Relationship Between Presence and Fear

Following the hypotheses of the study, ANOVAs were computed for both the presence and fear ratings with the presence manipulation (sensory realism) as between factor and the fear manipulation (situation) as within factor.

For a causal effect of fear → presence, we expected presence ratings to be higher in the height situation compared to the control situations. The ANOVA showed a significant main effect for the presence manipulation, *F*(1,46) = 5.70, *p* = 0.021, $\eta_{p}^{2}$ = 0.11, a significant main effect for the fear manipulation, *F*(1.73,79.40) = 13.01, *p* < 0.001, $\eta_{p}^{2}$ = 0.22, and no interaction effect, *F*(1.73,79.40) = 0.07, *p* = 0.905, $\eta_{p}^{2}$ < 0.01 (see Figure 3A and Supplementary Material). For the significant main effect for the presence manipulation, means indicate higher presence ratings in the high sensory realism compared to the low sensory realism condition. For the significant main effect for the fear manipulation, *post hoc* pairwise comparisons (alpha adjustment with Tukey’s method) between situations yield a significant difference between control situation 1 and the height situation, *t*(46) = −4.36, *p* < 0.001, a significant difference between the height situation and the control situation 2, *t*(46) = 3.43, *p* = 0.004, and no difference between control situation 1 and control situation 2, *t*(46) = −1.69, *p* = 0.220. Presence ratings in the height situation were higher than in both control situations.

For a causal effect of presence → fear, we expected fear ratings specifically in the height situation to be higher in the high sensory realism condition. The ANOVA on fear ratings revealed no main effect for the presence manipulation, *F*(1,47) = 1.02, *p* = 0.317, $\eta_{p}^{2}$ = 0.02, a significant main effect for the fear manipulation, *F*(1.10,51.62) = 161.63, *p* < 0.001, $\eta_{p}^{2}$ = 0.77, and no interaction effect, *F*(1.10,51.62) = 0.92, *p* = 0.350, $\eta_{p}^{2}$ = 0.02 (see Figure 3B and Supplementary Material). *Post hoc* pairwise comparisons (alpha adjustment with Tukey’s method) between situations yield a significant difference between control situation 1 and the height situation, *t*(47) = −12.86, *p* < 0.001, a significant difference between the height situation and the control situation 2, *t*(47) = 12.98, *p* < 0.001, and no difference between control situation 1 and control situation 2, *t*(94) = −1.18, *p* = 0.469. Fear ratings in the height situation were higher than in both control situations.

### Physiological Responses

Skin conductance and heart rate were analyzed analogously to the ratings. The ANOVA for SCL revealed no main effect for the presence manipulation, *F*(1,46) = 0.13, *p* = 0.717, $\eta_{p}^{2}$ < 0.01, a significant main effect for the fear manipulation, *F*(2,92) = 81.10, *p* < 0.001, $\eta_{p}^{2}$ = 0.64, and no interaction effect, *F*(2,92) = 0.31, *p* = 0.731, $\eta_{p}^{2}$ < 0.01 (see Figure 3C and Supplementary Material). *Post hoc* pairwise comparisons (alpha adjustment with Tukey’s method) between situations yield a significant difference between control situation 1 and the height situation, *t*(46) = −10.71, *p* < 0.001, a significant difference between the height situation and the control situation 2, *t*(46) = 11.06, *p* < 0.001, and no difference between control situation 1 and control situation 2, *t*(92) = −1.61, *p* = 0.251. SCL values in the height situation were higher than in both control situations.

For the heart rate, the ANOVA showed no main effect for the presence manipulation, *F*(1,47) = 0.92, *p* = 0.341, $\eta_{p}^{2}$ = 0.02, a significant main effect for the fear manipulation, *F*(1.32,61.99) = 7.97, *p* = 0.003, $\eta_{p}^{2}$ = 0.14, and no interaction, *F*(1.32,61.99) = 0.21, *p* = 0.713, $\eta_{p}^{2}$ < 0.01 (see Figure 3D and Supplementary Material). *Post hoc* pairwise comparisons (alpha adjustment with Tukey’s method) between situations yield a significant difference between control situation 1 and the height situation, *t*(47) = −3.05, *p* = 0.010, no difference between the height situation and the control situation 2, *t*(47) = 1.35, *p* = 0.377, and a significant difference between control situation 1 and control situation 2, *t*(47) = −4.07, *p* < 0.001. Heart rate in the height situation and control situation 2 were higher than in control situation 1.

### Exploratory Correlations and Cross-Lagged Panel Models

Two *post hoc* exploratory analyses were conducted to take a more in-depth look into the associations between presence and fear ratings.

First the bivariate correlation between presence and fear ratings in the height situation for the complete sample was, *r*(47) = 0.62, *p* < 0.001. Within the two groups varying in sensory realism (presence manipulation) the correlations were *r*(22) = 0.80, *p* < 0.001 for the group with high sensory realism and *r*(23) = 0.42, *p* = 0.038 for the group with low sensory realism, with a significant difference between these correlation coefficients, *z* = 2.13, *p* = 0.033.

**FIGURE 4:**
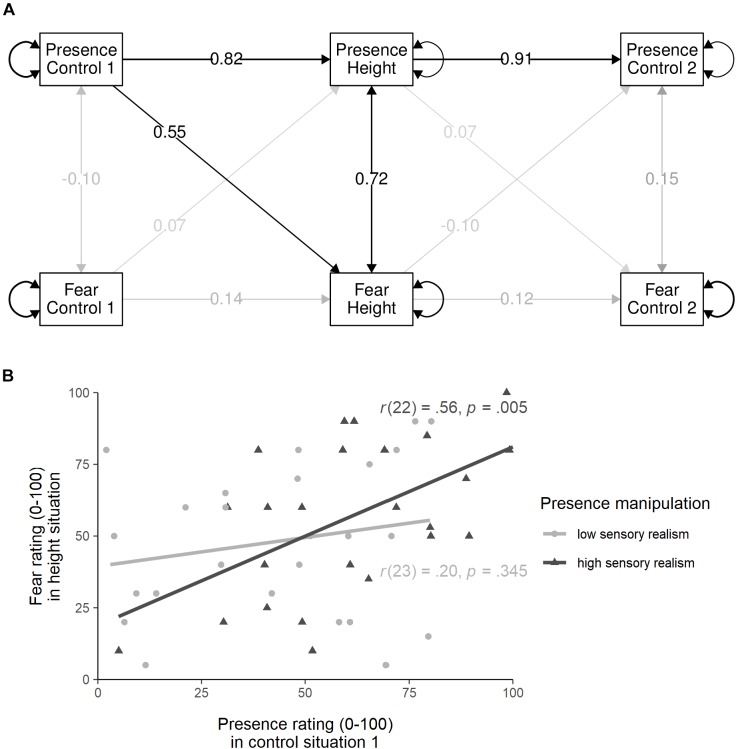
**(A)** Cross-lagged panel model for the high sensory realism condition with presence and fear ratings measured at three time points: control situation 1, height situation, and control situation 2. Black lines indicate significant paths of *p* < 0.01, gray lines indicate non-significant paths, and numbers display standardized path coefficients. **(B)** Scatter plot displaying the correlation between presence ratings in the control situation 1 and fear ratings in the height situation, differentiated for the two sensory realism conditions (presence manipulation).

Second, we fitted the presence and fear ratings in cross-lagged panel models, again split by the between-subject factor, to test whether presence and fear ratings would predict ratings in successive trials (see Peperkorn et al., 2015, for a similar, but correlational approach). In the high sensory realism group (see Figure 4A), significant paths were (1) the autoregressive paths for presence: presence in the height situation was predicted by presence in the control situation 1, β_std_ = 0.82, *p* < 0.001, and presence in the control situation 2 was predicted by presence in the height situation, β_std_ = 0.91, *p* < 0.001; (2) the regression coefficient of presence in the control situation 1 predicting fear in the height situation, β_std_ = 0.55, *p* = 0.002; and (3) the correlation between presence and fear in the height situation, *r* = 0.72, *p* = 0.005. In the low sensory realism group, only the autoregressive paths for presence were significant (both *p* < 0.001). For further visualization of the regression of initial presence ratings predicting later fear ratings in the height situation, the correlation between presence ratings in the first control situation and fear ratings in the height situation were calculated and are displayed in Figure 4B.

## Discussion

The present study investigated two research questions: first, whether a manipulation of sensory realism of a VE, i.e., high versus low quality of visual and auditory content, has an influence on experienced presence, and second, whether there is a causal relationship between presence and fear in VR. For this purpose, both presence and fear were manipulated experimentally in VR. Height-fearful participants were immersed into a virtual height situation and a control situation (fear manipulation) with either high or low sensory realism (presence manipulation). During immersion, we assessed ratings of presence and verbal and physiological fear responses.

### Effects of Sensory Realism on Presence

The use of highly detailed geometry, increased texture quality, and sound, compared to a low fidelity setup, led to increased presence ratings. This finding is in line with previous research (Hendrix and Barfield, 1996; Welch et al., 1996; Dinh et al., 1999; Larsson et al., 2007; Slater et al., 2009; Kwon et al., 2013; Brinkman et al., 2015), and the calculated effect size (small to medium) is in line with a recent meta-analysis (Cummings and Bailenson, 2016). Inconsistent with the verbal online ratings of presence assessed at the end of each VR experience, the presence questionnaire, measured after the experiment, revealed no difference between the two groups who experienced different sensory realism conditions. Earlier studies on the effects of the quality of visual and auditory content on presence have applied numerous presence measures (Cummings and Bailenson, 2016) and these diverse measures might differ in their sensitivity to detect an effect of manipulations of visual realism and auditory content on presence. Previous research suggests that such discrepancies between different measures of presence are not uncommon (Kober and Neuper, 2013). However, to our knowledge, there has not yet been an extensive comparison on such qualitative differences between multiple measures of presence. Another important point is that different presence measures also quantify different aspects of presence (e.g., spatial presence in the MEC-SPQ; spatial presence, involvement, and experienced realism in the Igroup Presence Questionnaire). The conviction of having been located in either the high or low sensory realism version of our VEs (spatial presence) might, in retrospect, not differ between groups, because both conditions allowed the same spatial perception of the environment. A manipulation of stereoscopy for example, which affects depth perception, might have had a stronger influence on retrospective reports of the experience of spatial presence (Cummings and Bailenson, 2016). In sum, we found some indication that our experimental manipulation of sensory realism modulated presence, although with a small to medium effect size only and restricted to one of two measures. Given this result, costly efforts to achieve a high sensory realism of VEs, e.g., by use of photogrammetry in the modeling process, might not be necessary for VEs to be plausible and to be able to induce presence. This argument is further supported by a comparison of presence responses to VEs across decades (e.g., Krijn et al., 2004; Gromer et al., 2018), where the 2004 study achieved even higher scores on the Igroup Presence Questionnaire^3^. However, it remains an open research question, whether VEs that achieved high presence in earlier studies, also induce high presence today (e.g., due to different standards of users). Further research is therefore needed to decide whether costly efforts to increase sensory realism are reasonable to increase presence.

### Effects of Fear on Presence

Our study expands previous research on the relationship between presence and fear in VR as our experimental fear manipulation caused an increased sense of presence. Similar effects of fear on presence have been shown for snake phobia by Bouchard et al. (2008), test anxiety by Alsina-Jurnet and Gutiérrez-Maldonado (2010), and spider phobia by Peperkorn et al. (2016), with higher presence ratings in fear-relevant versus neutral VEs or in phobic versus non-phobic participants. Our results corroborate these reports by demonstrating that experiencing emotional responses in VR leads to stronger feelings of actually being there in the VE (see also Riva et al., 2007). To explain these findings in a theoretical model, Diemer et al. (2015) postulated an interoceptive attribution model of presence which proposes two main factors that lead to higher presence ratings: immersion (i.e., technological characteristics of the VR system) and arousal. Our results fully support this model as presence was increased by both the manipulation of sensory realism (i.e., immersion), as well as an arousal manipulation (i.e., the height situation compared to the control situation elicited higher arousal as indicated by skin conductance). At a first glance, two comparable previous studies (Diemer et al., 2016; Gromer et al., 2018), which could not find differences in presence between high and low height-fearful participants after exposure to virtual height environments, seem to contradict this interpretation. However, Gromer et al. (2018) did not collect any physiological measure of arousal, and therefore these results allow no firm conclusions about the interoceptive attribution model. Diemer et al. (2016) measured skin conductance but revealed equal levels of physiological arousal in high and low height-fearful participants in the height situation. Consequently, these findings are still in line with the interoceptive attribution model, because it states presence as a function of arousal.

### Effects of Presence on Fear

Our study revealed no support of a causal effect of presence on fear as our experimental manipulation of presence did not lead to increased levels of fear in the virtual height situation. Several explanations have to be discussed. First, the strength of the fear response might not be dependent upon presence. If this is the case, putting much effort in creating highly realistic VEs is not necessary for virtual exposure as simpler VEs might be sufficient. Second, effects of presence on fear might only be observable if the manipulation of presence is strong enough. This argument receives support by a comparison of our study’s effect sizes for the manipulation of presence ($\eta_{p}^{2}$ = 0.11) and fear ($\eta_{p}^{2}$ = 0.77), suggesting that the effect of the presence manipulation was probably too small. Third, following the presence as a gateway hypothesis (Felnhofer et al., 2014), fear might not increase linearly with higher presence but rather a certain degree of presence is necessary to provoke fear responses (Bouchard et al., 2008). Once this threshold is reached, further increases in presence do not further affect fear responses. In our study, both the high and low sensory realism conditions might already have induced enough presence to pass this threshold, which then resulted in similar fear responses in both groups. However, contrary to the assumptions of the presence as a gateway hypothesis (with increasing presence, there is a plateau in fear responses), the correlation between presence and fear responses was much stronger in the high sensory realism condition. In order to experimentally test the different discussed explanations, future studies should use stronger presence manipulations and/or have experimental designs inducing multiple levels of presence to specifically test the predictions of the presence as a gateway hypothesis.

Interestingly, and in concordance with findings by Peperkorn et al. (2015), in the high sensory realism group initial ratings of presence in the first control situation were predictive of fear ratings in the later height situation, indicating an effect of interpersonal variability in presence on fear. Referring back to the interoceptive attribution model of presence by Diemer et al. (2015), which postulated presence as a function of immersion and arousal, our results also highlight the importance of user characteristics in the emergence of presence (IJsselsteijn et al., 2000; Wirth et al., 2007). User characteristics that have been thought to have an influence on presence include immersive tendencies (Witmer and Singer, 1998; Robillard et al., 2003; Murray et al., 2007; Phillips et al., 2012; Kober and Neuper, 2013; Ling et al., 2013), absorption (Baños et al., 1999; Schuemie et al., 2005; Murray et al., 2007; Phillips et al., 2012; Wirth et al., 2012; Kober and Neuper, 2013; Ling et al., 2013), dissociation (Baños et al., 1999; Murray et al., 2007; Phillips et al., 2012; Williams, 2014), spatial abilities (Alsina-Jurnet and Gutiérrez-Maldonado, 2010; Coxon et al., 2016), and personality (Alsina-Jurnet and Gutiérrez-Maldonado, 2010; Kober and Neuper, 2013). According to Kober and Neuper (2013), who studied the relationship between user characteristics and multiple presence measures, the best predictor for presence was absorption, followed by immersive tendencies, perspective taking, and mental imagination. Of note is that the relationship between interpersonal variability in presence in the neutral situation and later fear ratings in the height situation was only significant for the group with high visual realism and auditory content. Furthermore, the correlation between presence and fear, both measured in the height situation, was higher in the high than low sensory realism condition. Based on these findings, we suggest that it might be beneficial to use VEs with high sensory realism in studies on the presence-fear relationship.

### Limitations

Some limitations of the present study should be noted. First, as noted earlier, the effect of the presence manipulation, compared to the effect of the fear manipulation, was rather weak, possibly hindering a measurable influence of presence on fear. This was also reflected in a discrepancy between verbal presence ratings and presence measured via questionnaire. In future studies, stronger presence manipulations should be used to address this issue, e.g., by realizing various sensory realism manipulation plus manipulations in stereoscopy or user-tracking (Cummings and Bailenson, 2016).

Second, the cross-lagged panel model was conducted *post hoc* in an exploratory manner. To corroborate our findings, a further study should be planned and conducted with a priori hypotheses about the relationships within the cross-lagged panel model.

Third, the present study investigated only participants with a subclinical fear of heights. A recent meta-analysis revealed differences in the magnitude of the correlation between presence and fear between different phobias and between clinical and non-clinical fearful participants (Ling et al., 2014). It is therefore crucial to replicate the findings in other phobias, as well as a clinical population, with regards to generalizability.

## Conclusion

The present study sheds light on the causal interaction between presence and fear responses in VR and indicates a bidirectional relationship between both variables. First, our results show that experiencing fear as indicated by verbal and physiological responses in virtual heights leads to higher presence, supporting the hypothesis that arousal is an important factor in the formation of presence. Second, although our experimental manipulation of presence did not affect fear responses, the link between interpersonal variability in presence on the one hand and fear responses on the other hand suggests that higher presence leads to stronger fear responses. Furthermore, this finding stresses the importance to take user characteristics in the emergence of presence into account. Further studies are needed to test whether other experimental (e.g., different manipulations of immersion) or quasi-experimental manipulations (e.g., users with different characteristics) of presence have an influence on fear responses.

## Data Availability

The dataset for this study can be found at osf.io/8z6gt/.

## Author Contributions

DG, MR, IC, and PP contributed to the study concept and design. MR and IC collected the data. DG performed the data analysis and interpretation under the supervision of PP. DG drafted the article and MR, IC, and PP provided critical revisions. All authors approved the final version of the article prior to submission.

## Conflict of Interest Statement

PP is shareholder of a commercial company that develops virtual environment research systems (VTplus GmbH) for empirical studies in the field of psychology, psychiatry, and psychotherapy. The remaining authors declare that the research was conducted in the absence of any commercial or financial relationships that could be construed as a potential conflict of interest.

## Footnotes

1 https://github.com/dgromer/PeakMan

2 https://github.com/dgromer/phyr6

3 The Krijn et al. (2004) study used sums instead of means to calculate the total score of the Igroup Presence Questionnaire (IPQ). When scoring the data from Gromer et al. (2018) analogously, there is a significant difference between IPQ total scores, *t*(108) = 2.94, *p* = 0.004.
