# Supplementary material for: Causal Interactive Links Between Presence and Fear in Virtual Reality Height Exposure
Source: Front Psychol. 2019 Jan 30;10:141. doi: 10.3389/fpsyg.2019.00141 (PMC6363698; doi:10.3389/fpsyg.2019.00141)
Supplement: Supplementary file 1 [file Table_1.DOCX]

Supplementary Material

# Supplementary Tables

Table 1: Means and standard deviations of the MEC-SPQ by sensory realism conditions.

|  | **Low sensory realism** | |  | **High sensory realism** | |
| --- | --- | --- | --- | --- | --- |
| **MEC-SPQ Subscale** | ***M*** | ***SD*** |  | ***M*** | ***SD*** |
| **Attention Allocation** | 4.26 | 0.55 |  | 4.34 | 0.65 |
| **Spatial Situation Model** | 4.14 | 0.48 |  | 4.11 | 0.62 |
| **Spatial Presence: Self Location** | 3.87 | 0.56 |  | 3.98 | 0.90 |
| **Spatial Presence: Possible Actions** | 3.65 | 0.69 |  | 3.63 | 0.88 |
| **Suspension of Disbelief** | 2.94 | 0.92 |  | 3.21 | 1.14 |

Table 2: Means and standard deviations of the SSQ by sensory realism conditions.

|  | **Low sensory realism** | |  | **High sensory realism** | |
| --- | --- | --- | --- | --- | --- |
| **SSQ Subscale** | ***M*** | ***SD*** |  | ***M*** | ***SD*** |
| **Nausea** | 20.67 | 28.23 |  | 21.46 | 24.40 |
| **Oculomotor Problems** | 18.63 | 15.48 |  | 19.27 | 17.74 |
| **Disorientation** | 35.38 | 49.59 |  | 28.42 | 31.39 |
| **Total** | 26.80 | 30.26 |  | 25.56 | 24.15 |

Table 3: Means and standard deviations of the presence ratings by sensory realism conditions.

|  | **Low sensory realism** | |  | **High sensory realism** | |
| --- | --- | --- | --- | --- | --- |
|  | ***M*** | ***SD*** |  | ***M*** | ***SD*** |
| **Control situation 1** | 43.60 | 25.88 |  | 59.13 | 24.06 |
| **Height situation** | 54.60 | 22.63 |  | 70.22 | 24.05 |
| **Control situation 2** | 45.80 | 26.87 |  | 62.87 | 25.92 |

Table 4: Means and standard deviations of the fear ratings by sensory realism conditions.

|  | **Low sensory realism** | |  | **High sensory realism** | |
| --- | --- | --- | --- | --- | --- |
|  | ***M*** | ***SD*** |  | ***M*** | ***SD*** |
| **Control situation 1** | 2.08 | 3.80 |  | 3.75 | 8.63 |
| **Height situation** | 48.20 | 26.33 |  | 56.17 | 26.76 |
| **Control situation 2** | 4.12 | 6.17 |  | 4.00 | 12.23 |

Table 5: Means and standard deviations of baseline-corrected skin conductance level by sensory realism conditions.

|  | **Low sensory realism** | |  | **High sensory realism** | |
| --- | --- | --- | --- | --- | --- |
|  | ***M*** | ***SD*** |  | ***M*** | ***SD*** |
| **Control situation 1** | -0.02 | 0.05 |  | -0.02 | 0.07 |
| **Height situation** | 0.14 | 0.12 |  | 0.16 | 0.10 |
| **Control situation 2** | 0.01 | 0.10 |  | 0.00 | 0.08 |

Table 6: Means and standard deviations of baseline-corrected heart rate by sensory realism conditions.

|  | **Low sensory realism** | |  | **High sensory realism** | |
| --- | --- | --- | --- | --- | --- |
|  | ***M*** | ***SD*** |  | ***M*** | ***SD*** |
| **Control situation 1** | -3.99 | 3.84 |  | -3.12 | 3.75 |
| **Height situation** | -1.60 | 6.81 |  | 0.15 | 7.55 |
| **Control situation 2** | -2.18 | 3.68 |  | -1.15 | 4.87 |
